# Railway Surgeons

**Published:** 1904-07

**Authors:** C. A. Smith

**Affiliations:** Chief Surgeon; Tyler, Texas


### Correspondence.

### Railway Surgeons.

"Cotton Belt Route."
St. Louis Southwestern Railway Company—
Medical Department.
C. A. Smith, Chief Surgeon.

Tyler, Texas, May 16, 1904.

*Dr. F. E. Daniel, President State Medical Association of Texas, and Editor Texas Medical Journal.*

Dear Dr. Daniel: It is with pleasure that I acknowledge the receipt of your letter of May 12th, in which you tender me the Chairmanship of the New Section of Railway Surgery.

I accept the position and will do the very best I can to make the new section a success.

It has come to be already extensively recognized that railway surgery is a distinct specialty of the healing art, closely allied to military surgery. Large bodies of men are employed in the transportation service of the country, under a semi-military discipline, and the "hazard of the service" of these employes has for years attracted the attention of the thoughtful managers, both in this country and abroad.

Unfortunately the mechanical devices for the transportation of human freight come to grief and a calamity as terrible as a battle shocks the community.

Various systems have been adopted by the transportation companies tc meet these conditions, varging greatly in detail, but probably, on the whole, best adapted to the conditions of the section of country through which the lines run.

Most of the roads of the Southwest have adopted the hospital system as being best suited to a sparsely settled country with towns far apart and comparatively few local hospitals where the sick and injured can find suitable accommodations.

Questions can be discussed in this section, such as the education of employes in first aid to the injured, the transportation of the injured, medical and surgical supplies and devices for the equipment of trains and stations, sanitation and disinfection of railway coaches and stations, freight and baggage, now being so ably advocated by our efficient State Health Officer, Dr. Tabor, preparation for and transportation of dead bodies on railway trains and its relation to the public health.

Model railway hospitals and hospital organization, system of company surgeons along the lines, their relations and duties to the employes, the company and general public, surgical wounds peculiar to railway accidents including violent and accidental injuries inflicted by rapidly moving and ponderous machinery, the medicolegal side of railway accidents, including the so-called railway "neurosis," "railway spine," etc.

In my opinion, there is ample room for an interesting section of this kind in connection with the State Medical Association. I have never been much in favor of separate organizations of railway surgeons. It seems to me that if there is any knowledge to be gained from studying this class of cases, the whole of the profession should have a chance at it. Such a section need not interfere with the general surgical section in the least, and it is distinct enough to be put in a class by itself.

Thanking you again for the honor you have conferred on me by making me the chairman of the new section, and promising my best efforts to make the section a success, I remain, as ever,

Your friend,

C. A. SMITH.

[It will be remembered that in the April "Red Back" I advocated the organization of railway surgeons. See the article. The creation of a Section on Railway Surgery by the State Medical Association of Texas at the April meeting does away with the necessity of a separate organization. I take the liberty of publishing Dr. Smith's letter as showing the necessity of concerted action by railway medical officers.—EDITOR.]

